# Supplementary material for: Mimicry and law: Experiments in a natural setting of a law company
Source: PLoS One. 2023 Oct 12;18(10):e0292699. doi: 10.1371/journal.pone.0292699 (PMC10569552; doi:10.1371/journal.pone.0292699)

**S1 Appendix.
Mini meta-analysis with mixed mimicry condition**

We carried out a mini meta-analysis to investigate whether mimicked participants/clients are more likely to give power of attorney to a mimicking lawyer. A mini meta-analysis will be conducted using a small number of studies (*k* = 2) with small sample sizes (both *n* = 60).

The observed *OR*_logged_ estimates ranged from *Min.* = 1.24 to *Max.* = 1.86. The estimated average *OR*_logged_ based on the random-effects model was *OR*_logged_ *=* 1.53, 95% CI [0.76, 2.3]. Therefore, the average outcome differed significantly from zero *z* = 3.91, *p* < .001. According to the *Q*-test, the true outcomes appear to be homogeneous, *Q*(1) = 0.62, *p* = .430, *τ*^2^ = 0.00, *I*^2^ = 1%. A 95% prediction interval is given by 0.76 to 2.3. Hence, the average outcome is estimated to be positive, with the true outcomes being positive in both studies.

This mini meta-analysis indicates that a somehow mimicked participant/client has 4.27 (*OR* = exp(*OR*_logged_) = exp(1.53) = 4.62) times greater odds of signing a power of attorney than a participant/client who was not mimicked. Furthermore, the 95% confidence interval for *OR* [2.13, 19.75] does not contain the value of 1. This means that there is a high certainty that the relationship between somehow mimicking and signing a power of attorney is true and not due to chance. For more details, please see **Fig**.

**Fig.** Forest Plot of Meta-Analyzed Samples.


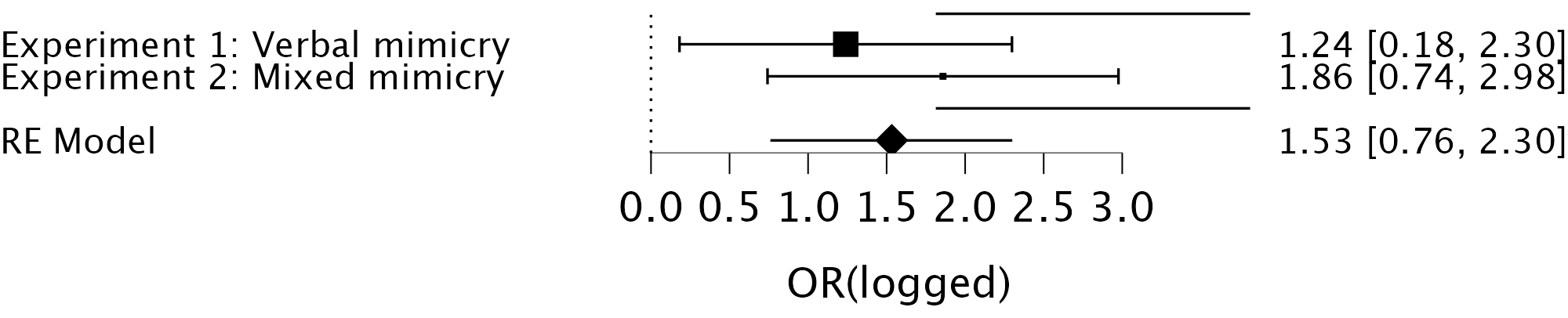

Supplement: S1 Appendix — (DOCX) [file pone.0292699.s001.docx]
