# Supplementary material for: Mimicry and law: Experiments in a natural setting of a law company
Source: PLoS One. 2023 Oct 12;18(10):e0292699. doi: 10.1371/journal.pone.0292699 (PMC10569552; doi:10.1371/journal.pone.0292699)
Supplement: Attachment — Submitted filename: Response_Letter_Revison.docx [file pone.0292699.s002.docx]

1. We suggest that you kindly consider reorganizing your manuscript and report using the 'IMRaD method', i.e. Introduction, Methods, Results , analysis and Discussion, before Limitations, etc. As it currently stands your manuscript includes multiple sections on procedures, results, and discussions. We feel reorganization of the manuscript will make for easier readability.

*Thank you for your valuable feedback. We appreciate your suggestion to reorganize the manuscript using the 'IMRaD' method. We understand the importance of a clear and logical structure for the readability of the manuscript.*

*In response to your comments, we have revised our manuscript to follow the 'IMRaD' structure, i.e., Introduction, Methods, Results, Analysis, and Discussion, before the Limitations section. We have consolidated the multiple sections on procedures, results, and discussions into these categories to provide a more streamlined and coherent presentation.*

*We believe these changes will significantly improve the readability and flow of our manuscript. Thank you again for your insightful suggestions. Please see p. 10 to p. 12.*

2. Also, at the end of your Methods section please include a sub-section on "ethical approvals", including information on methods of obtaining informed consent from participants and methods used to maintain data and participant confidentiality.

*Thank you for your comment. In accordance with your recommendation, the ethics statement has now been moved to the Methods sub-section “ethical approvals”.
It has been removed from all other sections.*

*Please see p. 12 to familiarize yourself with the changes.*

3. In your Introduction kindly expand briefly on the 'literature review with regards to trust building in legal contexts.'

*Thank you so much for this suggestion. The previous version of the manuscript clearly missed this important deliberation.*

*In the present version of the manuscript we have added the literature review on trust creation in the legal context (see p.3-4).*

*Thanks to this great comment we have - in the discussion section - also briefly described the input of our research to the body of literature on trust in legal contexts (see p.18).*

4. In your discussion kindly address the potential 'ethical considerations regarding the practical application of the results of your study, and also expatiate or reflect briefly 'on the effects of lawyers using mimicry in their interactions with clients.'

*Done! Please see p.23 – 24.*

5. Finally, in your abstract and discussion, and conclusions kindly refrain from to 'overinterpretation on the importance of the obtained results' from this study, as suggested by one reviewer.

*You are absolutely right! We were too “excited” while describing the results. In the present version of the manuscript we discuss (both: abstract and discussion) the results in a more restrained way.*

Please submit your revised manuscript by Sep 01 2023 11:59PM. If you will need more time than this to complete your revisions, please reply to this message or contact the journal office at plosone@plos.org. When you're ready to submit your revision, log on to https://www.editorialmanager.com/pone/ and select the 'Submissions Needing Revision' folder to locate your manuscript file.

Please include the following items when submitting your revised manuscript:

- A rebuttal letter that responds to each point raised by the academic editor and reviewer(s). You should upload this letter as a separate file labeled 'Response to Reviewers'.
- A marked-up copy of your manuscript that highlights changes made to the original version. You should upload this as a separate file labeled 'Revised Manuscript with Track Changes'.
- An unmarked version of your revised paper without tracked changes. You should upload this as a separate file labeled 'Manuscript'.

If you would like to make changes to your financial disclosure, please include your updated statement in your cover letter. Guidelines for resubmitting your figure files are available below the reviewer comments at the end of this letter.

If applicable, we recommend that you deposit your laboratory protocols in protocols.io to enhance the reproducibility of your results. Protocols.io assigns your protocol its own identifier (DOI) so that it can be cited independently in the future. For instructions see: https://journals.plos.org/plosone/s/submission-guidelines#loc-laboratory-protocols. Additionally, PLOS ONE offers an option for publishing peer-reviewed Lab Protocol articles, which describe protocols hosted on protocols.io. Read more information on sharing protocols at https://plos.org/protocols?utm_medium=editorial-email&utm_source=authorletters&utm_campaign=protocols.

We look forward to receiving your revised manuscript.
Kind regards,

Sylvester Chidi Chima, M.D., L.L.M, LLD.
Academic Editor
PLOS ONE

**Journal Requirements:**

When submitting your revision, we need you to address these additional requirements.

1. Please ensure that your manuscript meets PLOS ONE's style requirements, including those for file naming. The PLOS ONE style templates can be found at

<https://journals.plos.org/plosone/s/file?id=wjVg/PLOSOne_formatting_sample_main_body.pdf> and

<https://journals.plos.org/plosone/s/file?id=ba62/PLOSOne_formatting_sample_title_authors_affiliations.pdf>

*Thank you for your comment. We appreciate your guidance. We have thoroughly reviewed PLOS ONE's style requirements and have made sure that our manuscript, including the file naming, is in full compliance with them. We understand the importance of adhering to these standards for a smooth and efficient review process.*

2. Thank you for stating the following financial disclosure:

"The publication co-financed by the Ministry of Education and Science subsidies for maintaining and developing the didactic and research potential of the SWPS University of Social Sciences and Humanities (number: SUB/INTERDYSC/2019/12)."

Please state what role the funders took in the study. If the funders had no role, please state: "The funders had no role in study design, data collection and analysis, decision to publish, or preparation of the manuscript."

If this statement is not correct you must amend it as needed.

Please include this amended Role of Funder statement in your cover letter; we will change the online submission form on your behalf.

*Thank you for your attention to our financial disclosure. We would like to clarify that the funders had no role in the study design, data collection and analysis, decision to publish, or preparation of the manuscript. We also add this important information to the cover letter. We appreciate your diligence in ensuring the transparency of our work.*

3. Your ethics statement should only appear in the Methods section of your manuscript. If your ethics statement is written in any section besides the Methods, please move it to the Methods section and delete it from any other section. Please ensure that your ethics statement is included in your manuscript, as the ethics statement entered into the online submission form will not be published alongside your manuscript.

*The ethics statement has now been moved to the Methods sub-section “ethical approvals” in the manuscript. It has been removed from all other sections.*

*Please see p. 12 to familiarize yourself with the changes.*

4. Please upload a new copy of Figures 1 and 2 as the detail is not clear. Please follow the link for more information: https://blogs.plos.org/plos/2019/06/looking-good-tips-for-creating-your-plos-figures-graphics/" <https://blogs.plos.org/plos/2019/06/looking-good-tips-for-creating-your-plos-figures-graphics>

*Thank you for your valuable feedback! We have taken your comments into consideration and have updated our figures to enhance their clarity as per your suggestion. The revised figures are now included in the updated version of the manuscript.*

5. Please include captions for your Supporting Information files at the end of your manuscript, and update any in-text citations to match accordingly. Please see our Supporting Information guidelines for more information: <http://journals.plos.org/plosone/s/supporting-information>

*We have carefully revised the description of our Supporting Information files in accordance with the guidelines provided. The captions for the Supporting Information files have been added at the end of our manuscript, and in-text citations have been updated to match accordingly.*

*Please see p. 16 and p. 33.*

6. Please review your reference list to ensure that it is complete and correct. If you have cited papers that have been retracted, please include the rationale for doing so in the manuscript text, or remove these references and replace them with relevant current references. Any changes to the reference list should be mentioned in the rebuttal letter that accompanies your revised manuscript. If you need to cite a retracted article, indicate the article’s retracted status in the References list and also include a citation and full reference for the retraction notice.

*Thank you very much for the call to action in this regard. The reference list has been verified as indicated. It is correct and complete. The referenced documents contained in the manuscript are authoritative. In addition, we inform you that the changes that have been made include the order of citations, caused by the introduction of corrections corresponding to the reorganization of the manuscript and report using the "IMRaD" method.*

**Reviewers' comments:**

Reviewer's Responses to Questions

**Comments to the Author**

1. Is the manuscript technically sound, and do the data support the conclusions?

The manuscript must describe a technically sound piece of scientific research with data that supports the conclusions. Experiments must have been conducted rigorously, with appropriate controls, replication, and sample sizes. The conclusions must be drawn appropriately based on the data presented.

Reviewer #1: Yes

Reviewer #2: Yes

2. Has the statistical analysis been performed appropriately and rigorously?

Reviewer #1: Yes

Reviewer #2: Yes

3. Have the authors made all data underlying the findings in their manuscript fully available?

The [PLOS Data policy](http://www.plosone.org/static/policies.action#sharing) requires authors to make all data underlying the findings described in their manuscript fully available without restriction, with rare exception (please refer to the Data Availability Statement in the manuscript PDF file). The data should be provided as part of the manuscript or its supporting information, or deposited to a public repository. For example, in addition to summary statistics, the data points behind means, medians and variance measures should be available. If there are restrictions on publicly sharing data—e.g. participant privacy or use of data from a third party—those must be specified.

Reviewer #1: Yes

Reviewer #2: Yes

4. Is the manuscript presented in an intelligible fashion and written in standard English?

PLOS ONE does not copyedit accepted manuscripts, so the language in submitted articles must be clear, correct, and unambiguous. Any typographical or grammatical errors should be corrected at revision, so please note any specific errors here.

Reviewer #1: Yes

Reviewer #2: Yes

**5. Review Comments to the Author**

Please use the space provided to explain your answers to the questions above. You may also include additional comments for the author, including concerns about dual publication, research ethics, or publication ethics. (Please upload your review as an attachment if it exceeds 20,000 characters)

Reviewer #1: Many thanks for this fascinating contribution. Mimicry among lawyers is, arguably, not studied at all, while it can contribute to successful negotiation strategies and trust building. I'm surprised you're not publishing this study in a more law-oriented journal, but PLOS One, being a prime generalist journal, should fit just as well.

My only minor comment is that you should expand a bit the lit review with regard to trust building in legal contexts, as you focus on psychology mainly.

*Done! Please see the corresponding reply above in this response letter (see p. 3-4)*

Reviewer #2: I liked the article for several reasons. First, it describes a series of studies carried out in natural conditions, which is increasingly rare in psychology. Secondly, although the research consists of field studies, it is well-prepared. The sample sizes are carefully calculated using the G*Power program, and the statistical analyses are conducted properly and sensibly. Both studies form a series, where minor errors made in the first study are fixed in the second. Furthermore, the authors also perform a mini meta-analysis to present the combined results of both experiments.

However, the article does have minor drawbacks that, in my opinion, require improvement. The authors tend to overinterpret the importance of the obtained results to some extent. In certain cases, certain adjectives seem redundant, such as in the abstract, where the phrase 'great potential' appears rather overly optimistic to me. Additionally, the authors allocate too little space to the ethical considerations regarding the practical application of their results. I feel that the article lacks reflection on the effects of lawyers using mimicry in their interactions with clients. Can a profession based on social trust employ social influence tools to manipulate their clients? It seems that this issue is not given sufficient attention in the article.

*Done!*

*We fully agree with your great point! Please see p.23 - 24.*

Despite the mentioned faults, I find the submitted article interesting and believe it is worth publishing in PlosOne after implementing the necessary corrections.

*****

*Finally, once again we would like to express our gratitude to the Reviewers for their input and insight which - we hope - improved our manuscript.*

6. PLOS authors have the option to publish the peer review history of their article ([what does this mean?](https://journals.plos.org/plosone/s/editorial-and-peer-review-process#loc-peer-review-history)). If published, this will include your full peer review and any attached files.


If you choose “no”, your identity will remain anonymous but your review may still be made public.


**Do you want your identity to be public for this peer review?** For information about this choice, including consent withdrawal, please see our [Privacy Policy](https://www.plos.org/privacy-policy).

Reviewer #1: No

Reviewer #2: No

[NOTE: If reviewer comments were submitted as an attachment file, they will be attached to this email and accessible via the submission site. Please log into your account, locate the manuscript record, and check for the action link "View Attachments". If this link does not appear, there are no attachment files.]

While revising your submission, please upload your figure files to the Preflight Analysis and Conversion Engine (PACE) digital diagnostic tool, <https://pacev2.apexcovantage.com/>. PACE helps ensure that figures meet PLOS requirements. To use PACE, you must first register as a user. Registration is free. Then, login and navigate to the UPLOAD tab, where you will find detailed instructions on how to use the tool. If you encounter any issues or have any questions when using PACE, please email PLOS at figures@plos.org. Please note that Supporting Information files do not need this step.
